# From Inflammation to Bone Loss: The Multifaceted Role of Neutrophils in Osteoporosis

**DOI:** 10.1155/ijin/4867595

**Published:** 2025-12-08

**Authors:** Hao Cheng, Yipeng Cheng, Guodong Wang, Decheng Wang, Ximing Liu

**Affiliations:** ^1^ Hubei Key Laboratory of Tumor Microenvironment and Immunotherapy, College of Basic Medical Sciences, China Three Gorges University, Yichang 443002, China, ctgu.edu.cn; ^2^ Department of Orthopedics, General Hospital of Central Theater Command of PLA, Wuhan, China; ^3^ Medical College, Wuhan University of Science and Technology, Wuhan, China, wust.edu.cn

**Keywords:** immune cell, neutrophils, osteoimmunology, osteoporosis

## Abstract

Neutrophils, the most abundant innate immune cells, have recently emerged as central regulators in the pathogenesis and treatment of osteoporosis. Traditionally viewed as transient inflammatory responders, neutrophils are now recognized as dynamic mediators linking immune dysregulation, bone remodeling, and aging in the context of osteoimmunology. This review provides a comprehensive synthesis of their multifaceted roles in bone metabolism: promoting osteoclastogenesis via RANKL, ROS, and neutrophil extracellular traps (NETs), while concurrently inhibiting osteoblast activity through TGF‐β1 and bone marrow‐derived stress signals. We highlight the involvement of neutrophils in postmenopausal osteoporosis driven by estrogen deficiency and in senile osteoporosis associated with immune aging, with particular attention to pathogenic subsets such as TGF‐β1^+^CCR5^+^ neutrophils. In addition, we examine the clinical relevance of neutrophil‐related biomarkers, such as neutrophil‐to‐lymphocyte ratio (NLR), systemic immune–inflammation index (SII), and citrullinated histone H3 (Cit‐H3), for diagnosis and risk stratification, as well as emerging therapeutic strategies that target NETosis and CCR5 signaling or employ neutrophil‐homing drug delivery systems. By elucidating these immune–bone interactions, neutrophils were found to be promising diagnostic biomarkers and immunotherapeutic targets in osteoporosis, paving the way for precision intervention.

## 1. Introduction

### 1.1. Epidemiology of Osteoporosis

Osteoporosis is a systemic bone disease characterized by low bone mass and deterioration of bone microarchitecture, leading to increased bone fragility and fracture risk, especially of the hip, spine, and wrist [1]. Approximately 200 million people suffer from osteoporosis worldwide, and the prevalence of osteoporosis is expected to increase by 310% in men and 240% in women by 2050 [2, 3]. The condition is especially common in postmenopausal women and the elderly. The global prevalence of osteoporosis is about 18.3%, including 11.7% in men and 23.1% in women [4]. Studies show that among individuals aged 50 and above, roughly one‐third of women and one‐fifth of men will experience an osteoporotic fracture [5]. These fractures not only significantly reduce the quality of life of patients but also increase the risk of disability and mortality, imposing a heavy socioeconomic burden [5, 6].

### 1.2. Traditional Pathogenesis of Osteoporosis

**Figure 1:**
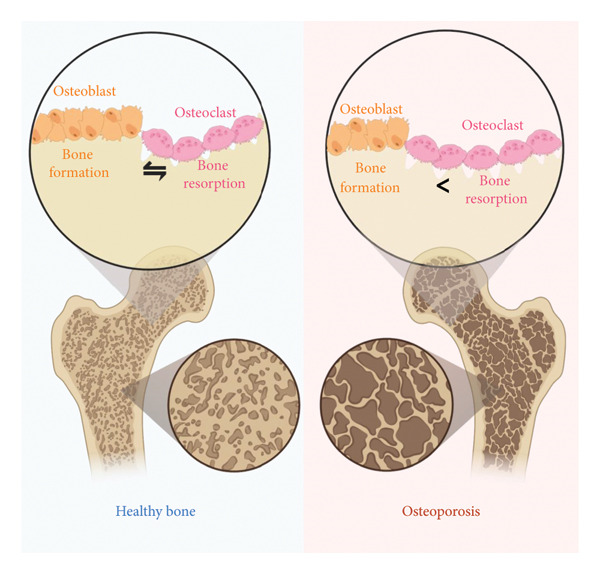
Traditional pathogenesis of osteoporosis. Osteoporosis is primarily caused by an imbalance in bone remodeling, where excessive osteoclast activation leads to bone resorption that surpasses bone formation, resulting in net bone loss. The figure was made on biorender.com.

The development of osteoporosis is primarily related to an imbalance in bone remodeling [7]. Under normal physiological conditions, osteoblasts and osteoclasts work together to maintain bone homeostasis [7]. In osteoporosis, however, osteoclast‐mediated bone resorption is enhanced while osteoblast‐mediated bone formation is reduced, resulting in net bone loss and deterioration of bone structure [8]. Estrogen deficiency is the main cause of postmenopausal osteoporosis (PMO) in women [9]. It accelerates bone loss by disrupting the balance between osteoblasts and osteoclasts [9]. Specifically, reduced estrogen levels prevent osteoclast apoptosis, enhance the receptor activator of nuclear factor‐κB ligand (RANKL)/OPG signaling pathway, and increase the secretion of proinflammatory cytokines (such as TNF‐α and IL‐6), thereby promoting osteoclast differentiation and activity and increasing bone resorption [9, 10]. Meanwhile, estrogen deficiency also suppresses the differentiation of mesenchymal stem cells (MSCs) into osteoblasts, decreases osteoblast survival, and reduces the synthesis of type I collagen, ultimately limiting bone formation [11, 12]. The synergy of these effects disrupts bone metabolic balance, leading to decreased bone density and the development of osteoporosis. In addition, aging is a significant risk factor for osteoporosis in the elderly: aging results in a decline in the number and osteogenic potential of bone marrow MSCs, which differentiate into osteoblasts under specific conditions, thereby reducing bone formation [13] (Figure 1).

### 1.3. Osteoimmunology and Neutrophils in Osteoporosis

In 2000, Arron and Choi first proposed the term “osteoimmunology” to describe the interface between the bone and immune systems [14]. Interactions between these systems have drawn interest in bone diseases such as rheumatoid arthritis (RA) [15], fracture healing [16], and osteoinduction [17]. Osteoporosis is a multifactorial disease associated with endocrine, metabolic, and mechanical factors. In recent years, studies have found that chronic inflammation can inhibit bone turnover and induce osteoporosis [18]. A low level of chronic inflammation occurs during aging (known as “inflammaging”) and is linked to the pathogenesis of osteoporosis [19]. Similarly, in postmenopausal women, estrogen deficiency leads to increased production of inflammatory cytokines, contributing to osteoporosis [10, 20]. The bone marrow constitutes a highly dynamic and complex microenvironment enriched with diverse immune and stromal cell populations. This cellular niche, including osteocytes, immune cells, and mesenchymal stromal cells, plays a critical role in maintaining skeletal homeostasis [21]. Increasing evidence has highlighted the bidirectional crosstalk between the skeletal and immune systems, where disruption of this delicate balance contributes substantially to the pathogenesis of osteoporosis. This concept has led to the emergence of the term “immunoporosis,” underscoring the immunological basis of bone loss [21, 22]. Bone marrow‐resident immune cells include macrophages, neutrophils, T cells, and B cells [21]. While previous studies have largely concentrated on the roles of macrophages [23], T lymphocytes, and B lymphocytes [24] in bone remodeling, neutrophils, despite being the most abundant leukocyte subset in the bone marrow, have only recently garnered attention as pivotal regulators of bone metabolism. Neutrophils not only coordinate the early immune response and recruit stromal and adaptive immune cells during bone injury and repair [25] but also directly modulate osteoclastogenesis, osteoblast inhibition, and MSC function in both physiological and pathological contexts [26].

This review aims to summarize the mechanisms by which neutrophils influence bone metabolism and drive bone loss to explore the potential of neutrophils as diagnostic indicators in osteoporosis and to discuss emerging therapeutic strategies targeting neutrophils (including immunomodulatory therapies, novel drug delivery systems, and anti‐inflammatory interventions). By integrating mechanistic insights with translational perspectives, this review aims to provide a solid framework for future basic research and clinical innovations in osteoimmunology and osteoporosis management.

## 2. Neutrophils and Bone Homeostasis: Mechanistic Insights

### 2.1. Origin and Basic Functions of Neutrophils

Neutrophils are immune cells derived from hematopoietic stem cells in the bone marrow and belong to the myeloid lineage of leukocytes [27, 28]. During hematopoiesis, they undergo a series of differentiation stages, including myeloid progenitor cells, granulocyte‐monocyte progenitor cells, myeloblasts, promyelocytes, myelocytes, metamyelocytes, and band cells, eventually maturing into segmented neutrophils characterized by multilobed nuclei [29]. Once mature, neutrophils are released into the peripheral blood and can rapidly migrate to sites of infection or inflammation in response to external stimuli, where they play a central role in innate immune defense [27, 28]. As the most abundant type of leukocytes in the innate immune system, neutrophils constitute approximately 40%–60% of circulating white blood cells and are essential for host defense [30, 31]. Their primary functions include phagocytosis of pathogens, degranulation, production of reactive oxygen species (ROS), and the release of neutrophil extracellular traps (NETs) [31, 32]. In acute inflammation, neutrophils are the first immune cells recruited to sites of infection or injury. Their mobilization from the bone marrow into circulation is regulated by granulocyte colony‐stimulating factor (G‐CSF) and chemokines such as CXCL8 (also known as IL‐8) [31]. Upon reaching the target tissue, neutrophils exert their antimicrobial effects through phagocytosis and the release of proteolytic enzymes, including elastase and myeloperoxidase, which can degrade microbial components [33] (Figure 2).

Figure 2The origin and functional roles of neutrophils. (a) Neutrophils originate from HSCs and differentiate through several stages in the bone marrow, ultimately forming mature neutrophils [29]. (b) Mature neutrophils perform essential immune functions, including phagocytosis, reactive oxygen species (ROS) generation, degranulation, and the formation of neutrophil extracellular traps (NETs), thereby playing a central role in antimicrobial defense and inflammation [32]. HSC: hematopoietic stem cell; MPP: multipotent progenitor; LPMP: lymphoid‐primed multipotent progenitors; GMP: granulocyte‐monocyte progenitors.

**Figure:**
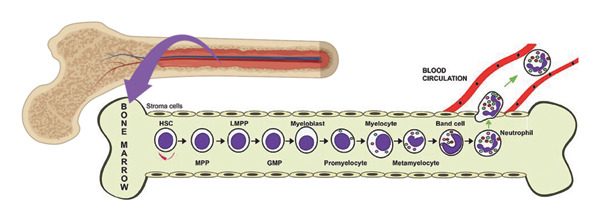
(a)

**Figure:**
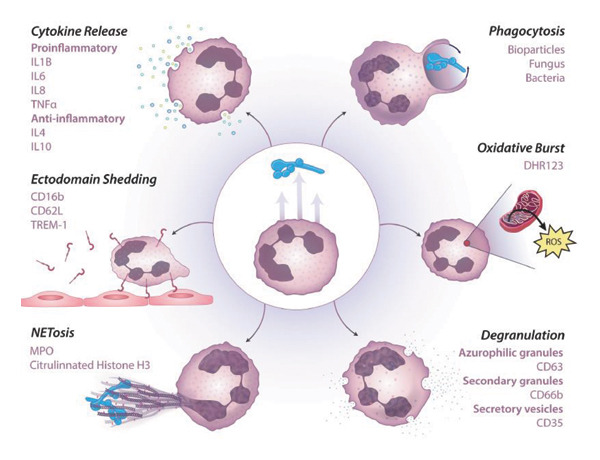
(b)

Beyond direct pathogen clearance, neutrophils also produce cytokines and chemokines in response to local inflammatory signals, thereby modulating other immune and inflammatory cells [34]. Although their lifespan is relatively short, typically 6–8 h in circulation, it can be prolonged under conditions of chronic inflammation or immune dysregulation, contributing to sustained tissue damage and prolonged inflammatory responses [35]. Moreover, neutrophils exhibit a proinflammatory phenotype under conditions such as immunosenescence and estrogen deficiency, which has been associated with enhanced bone resorption and increased risk of osteoporosis [26, 36]. Therefore, beyond their traditional immune defense function, neutrophils also play a critical role in regulating bone metabolism, immune‐inflammatory balance, and the development of osteoporosis [37].

### 2.2. Neutrophil‐Mediated Osteoclastogenesis

#### 2.2.1. Neutrophil‐Derived RANKL Signaling in Osteoclastogenesis

Neutrophils promote osteoclastogenesis primarily through the RANKL signaling axis [26, 36]. Activated neutrophils express membrane‐bound RANKL (mRANKL) on their surface, which binds to RANK receptors on osteoclast precursors, driving their differentiation and activation [36, 38]. In inflammatory diseases such as RA and chronic obstructive pulmonary disease (COPD), the number of RANKL‐positive neutrophils in the blood and synovial fluid increases significantly, which correlates with reduced bone mineral density (BMD) [36]. Moreover, activation of toll‐like receptor 4 (TLR4) on neutrophils can upregulate mRANKL expression, further enhancing osteoclast activity and accelerating bone resorption [36, 38]. In addition to direct RANKL expression, neutrophils secrete proinflammatory cytokines such as IL‐1β, TNF‐α, and IL‐17 that stimulate RANKL production by bone marrow stromal cells and other immune cells [36, 39]. In particular, IL‐1β not only induces RANKL expression but also stimulates the release of G‐CSF, thereby increasing neutrophil mobilization and further amplifying inflammation and bone resorption [39].

#### 2.2.2. Neutrophil‐Derived ROS Promote Osteoclastogenesis

The respiratory burst of activated neutrophils produces abundant ROS via NADPH oxidase, which can directly promote the differentiation and maturation of osteoclast precursors through oxidative signaling mechanisms [26]. Moreover, ROS activates the nuclear factor of activated T‐cells c1 (NFATc1) pathway, further enhancing osteoclast differentiation and contributing to osteoporosis [26]. Additionally, RANKL stimulation itself enhances ROS generation, creating a positive feedback loop that further drives osteoclast formation [26]. In conditions such as estrogen deficiency, neutrophil hyperactivation leads to excessive ROS accumulation, exacerbating bone resorption and contributing to osteoporosis progression [9, 24].

#### 2.2.3. Neutrophil‐Derived NETs in Osteoclast Activation

Neutrophils undergoing NETosis release NETs composed of DNA, histones, and granule proteins [39, 40]. NETs directly activate TLR4/TLR9 pathways on osteoclast precursors, stimulating their differentiation into mature osteoclasts [39, 40]. NETs also activate the NLRP3 inflammasome, resulting in elevated secretion of IL‐1β and upregulation of RANKL, which together amplify osteoclastogenic signaling [39, 40]. Furthermore, ROS produced during NET formation activate the MAPK/ERK signaling cascade, further enhancing osteoclast functional activity and bone resorption [39–41].

#### 2.2.4. Emerging Neutrophil‐Derived Mechanisms Promoting Osteoclastogenesis

**Figure 3:**
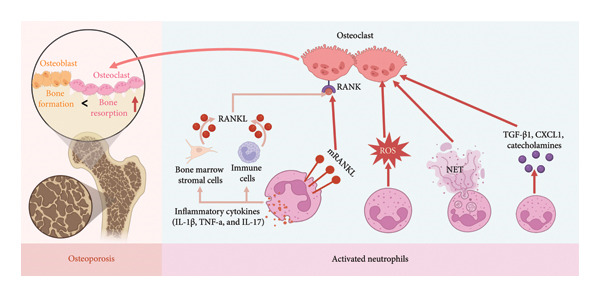
Neutrophil‐mediated mechanisms of osteoclastogenesis. Neutrophils promote osteoclast activation through multiple pathways, including direct membrane‐bound RANKL (mRANKL)–RANK interaction, reactive oxygen species (ROS) generation, NET‐induced signaling, and the secretion of osteoclastogenic mediators such as TGF‐β1, CXCL1, and catecholamines. The figure was made on biorender.com.

In addition to classical inflammatory mechanisms, several emerging neutrophil‐derived pathways contribute to osteoclastogenesis. Expansion of TGF‐β1⁺CCR5⁺ neutrophils (TCNs) in the bone marrow of aged mice enhances osteoclast activity through local TGF‐β1 signaling [19]. Moreover, neutrophil‐secreted CXCL1 binds to CXCR2 receptors on osteoclast precursors, promoting their maturation and accelerating bone resorption [42]. Under chronic stress, neutrophils upregulate tyrosine hydroxylase (TH) and release catecholamines (CAs) such as norepinephrine, which further stimulate osteoclastogenesis via *β*‐adrenergic receptor‐mediated signaling [43] (Figure 3).

### 2.3. Neutrophil‐Mediated Inhibition of Osteoblast Function

#### 2.3.1. Neutrophil‐Derived TGF‐β1 and Inhibition of Osteoblast Activity

Expansion of TCN subsets in the bone marrow leads to continuous TGF‐β1 secretion, suppressing osteoblast function [19]. In osteoporosis, this mechanism reduces osteogenic activity and increases bone resorption, accelerating bone loss [44].

#### 2.3.2. Neutrophil‐Derived NETs and Osteoblast Apoptosis

NET formation not only enhances the local inflammatory environment [40] but also directly induces osteoblast apoptosis via the release of extracellular DNA, elastase, and myeloperoxidase [45]. Excessive NET formation in osteoporosis patients leads to persistent bone microenvironment damage and impairs bone repair [40, 41].

#### 2.3.3. Neutrophil‐Derived ROS and Suppression of Osteoblast Differentiation

**Figure 4:**
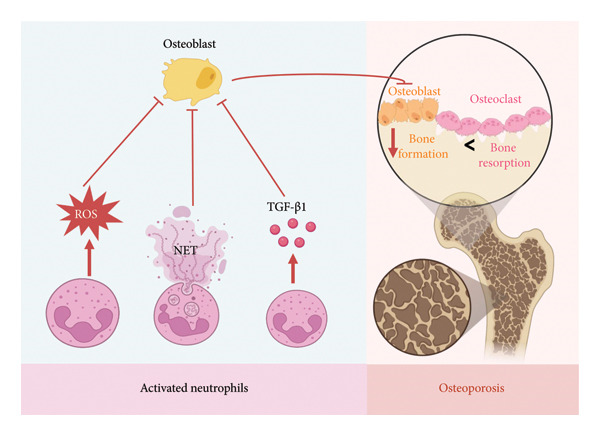
Neutrophil‐derived mechanisms inhibiting osteoblast function. Activated neutrophils impair osteoblast activity through excessive ROS production, NET‐induced cytotoxicity, and secretion of inhibitory cytokines such as TGF‐β1. The figure was made on biorender.com.

Activated neutrophils produce abundant ROS, which downregulate key osteogenic transcription factors such as Runx2 and Osterix, thereby inhibiting osteoblast differentiation and matrix mineralization [26]. This oxidative stress‐mediated suppression of osteoblast function further contributes to compromised bone formation in osteoporosis (Figure 4).

### 2.4. Neutrophil‐Mediated Regulation of MSC Fate

While neutrophils impair mature osteoblast function, they also profoundly influence bone homeostasis by regulating MSC fate at earlier stages of osteogenic commitment.

#### 2.4.1. Neutrophil‐Derived TGF‐β1 and Suppression of MSC Osteogenic Differentiation

**Figure 5:**
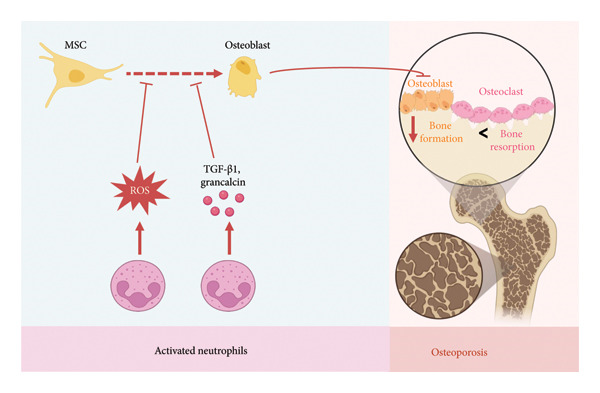
Neutrophil‐mediated regulation of mesenchymal stem cell (MSC) fate. Neutrophil‐derived factors, including TGF‐β1, ROS, and grancalcin (GCA), suppress MSC osteogenic differentiation, induce MSC apoptosis, and promote adipogenic transdifferentiation, thereby impairing bone formation. The figure was made on biorender.com.

In the setting of aging and chronic inflammation, TCNs expand in the bone marrow and secrete high levels of TGF‐β1, significantly inhibiting the differentiation of mesenchymal progenitor cells (MPCs) into osteoblasts [19]. This shift contributes to reduced bone formation and enhanced bone resorption (Figure 5).

#### 2.4.2. Neutrophil‐Derived ROS and Induction of MSC Apoptosis

Neutrophil‐generated ROS not only impair MSC differentiation into osteoblasts but also directly induce apoptosis of MSCs and osteoblasts, particularly under G‐CSF‐induced neutrophil proliferation [26].

#### 2.4.3. Neutrophil‐Derived Grancalcin (GCA) and Inhibition of Osteogenesis

Accumulation of proinflammatory neutrophils results in elevated secretion of GCA in bone marrow [46]. GCA binds to the PLXNB2 receptor on bone marrow‐MSCs, inhibiting the FAK‐YAP signaling pathway, thereby suppressing osteogenesis and promoting adipogenesis [46].

#### 2.4.4. Neutrophil‐Derived CAs and Stress‐Induced MSC Dysfunction

Under chronic stress, neutrophils express TH and release CAs such as norepinephrine [43]. CAs inhibit the transdifferentiation of chondrocytes to osteoblasts via activation of *α*/*β*_2_‐adrenergic receptors on chondrocytes, while also promoting inflammatory chemokine production (e. g., CXCL1), aggravating bone marrow inflammation and bone resorption [43]. *β*_2_‐Adrenergic receptor blockade has shown promise in mitigating these effects and improving bone quality.

## 3. Neutrophils in PMO: Immune Amplifiers of Bone Loss

PMO is characterized by decreased bone mass and increased fracture risk, primarily caused by the dramatic drop in estrogen levels after menopause [24, 47]. Estrogen directly promotes the differentiation of MSCs into osteoblasts and induces osteoclast apoptosis, thereby enhancing bone formation and limiting bone resorption [9]. In addition to these direct skeletal effects, estrogen deficiency also alters the immune status in postmenopausal women, which can lead to ongoing bone destruction. Postmenopausal women typically exhibit a chronic low‐grade inflammatory phenotype, with altered cytokine expression profiles and immune cell populations [9, 24]. Studies have shown that estrogen deficiency prolongs neutrophil lifespan, enhances neutrophil chemotaxis, and significantly increases NET formation [9, 20, 24]. In PMO models (such as ovariectomized mice), bone marrow neutrophils exhibit markedly upregulated NETosis, and the NETs can drive macrophage M1 polarization, induce osteoclast formation, and enhance bone resorption through the cGAS‐STING‐AKT2 pathway, identifying a novel therapeutic target for PMO [20]. In the absence of estrogen, excessive neutrophil activation also causes osteoblast apoptosis through ROS release and increases osteoclastogenesis via RANKL signaling [24].

In addition, clinical studies have shown that the neutrophil/lymphocyte ratio (NLR) in postmenopausal women is significantly increased and negatively correlates with BMD, making it a convenient indicator of systemic inflammation and bone loss risk [47]. Some studies have also found that selective estrogen modulators such as raloxifene can downregulate NET formation, thereby slowing the process of bone loss [9]. In summary, neutrophils serve a key immunoregulatory role in PMO by enhancing osteoclastogenesis, inhibiting osteoblast function, and sustaining a proinflammatory environment. They form a crucial link in the pathological chain of “estrogen deficiency‐immune activation‐bone remodeling imbalance” and offer potential targets for future immunomodulatory therapy.

## 4. Neutrophils in Immune Aging and Senile Osteoporosis

With advancing age, the immune system gradually declines, a process known as immunosenescence [26]. As key cells of innate immunity, neutrophils undergo significant functional and phenotypic changes with age. Immunosenescent neutrophils are characterized by reduced apoptosis, weakened adhesion and chemotaxis, impaired phagocytosis, and abnormal ROS release [18, 26]. In the bone marrow environment, neutrophils with blocked apoptosis progressively accumulate and continuously release ROS, inflammatory cytokines, and NETs, causing increased oxidative stress and inflammatory activation in the bone tissue microenvironment [18, 19, 26].

Studies have found that aging neutrophils are marked by an increased proportion of GCA‐positive (GCA^+^) cells. These GCA^+^ neutrophils inhibit bone formation, promote bone marrow fat accumulation, and reduce the bone turnover rate by releasing GCA, thereby accelerating skeletal aging and osteoporosis [46]. In addition, the TCN subsets significantly accumulate in the bone marrow of elderly individuals, with enhanced RANKL signaling and osteoclastogenesis and a suppressive effect on MSC osteogenesis, making them key drivers of age‐related bone loss [19]. Moreover, a CXCL8^+^ neutrophil subset is significantly enriched in aged individuals and is strongly associated with osteoclast differentiation pathways and inflammatory networks (such as those in RA), ultimately promoting bone loss and osteoporosis [21].

In summary, neutrophils in the immunosenescent state are altered in number, function, and secretory profile, becoming common promoters of osteoclast activation, osteoblast inhibition, and bone marrow fat accumulation in the aging skeleton. They represent an important immune mechanism underlying osteoporosis progression in the elderly. Targeting neutrophil‐associated pathways such as GCA or TGF‐β1/CCR5 to modulate neutrophil function may become a novel strategy for the treatment of senile osteoporosis in the future.

## 5. Diagnostic Potential: Neutrophil‐Derived Inflammatory Indices and NET Markers

In recent years, neutrophils, as core cells in the bone immune regulatory network, have been found to play a role in osteoporosis pathogenesis and to possess potential diagnostic value. Inflammation‐related blood indices are simple and cost‐effective biomarkers that are widely used to reflect the level of systemic inflammation. Among them, neutrophil‐based inflammatory indices, including NLR, SII (defined as platelets × neutrophils/lymphocytes), systemic inflammatory response index (SIRI, neutrophils × monocytes/lymphocytes), and the aggregate index of systemic inflammation (AISI, neutrophils × monocytes × platelets/lymphocytes), have been strongly associated with fracture risk and mortality in patients with PMO and osteopenia [48, 49].

### 5.1. NLR and Systemic Inflammatory Indices

NLR and SII are currently the most commonly examined inflammatory markers in osteoporosis research. NLR is significantly higher in osteoporotic patients and shows a negative correlation with BMD [2, 50, 51]. One study reported that an NLR above 2.02 is associated with a markedly increased risk of osteoporosis, suggesting NLR as a convenient and sensitive indicator for assessing inflammatory risk in clinical evaluations [52]. Moreover, elevated NLR has been identified as an independent risk factor for PMO [53], osteoporotic vertebral fractures [54], and femoral neck fractures [55] and even for osteoporosis in patients with RA [56]. In postmenopausal women with type 2 diabetes, NLR has also been found to predict osteoporosis risk [57]. The SII, a composite index integrating neutrophil, lymphocyte, and platelet counts, is significantly correlated with bone loss [51, 58] and has been used to predict the risk of PMO [58–60], senile osteoporosis [61], and osteoporotic fractures [59]. From a clinical perspective, some research suggests that SII may be a better inflammatory marker for predicting osteopenia and osteoporosis in postmenopausal women ≥ 50 years old [60].

### 5.2. NET Markers

In addition to blood cell ratios, NETs formed during neutrophil activation also offer important diagnostic potential. Components of NETs, such as citrullinated histone H3 (Cit‐H3), are elevated in the serum of osteoporosis patients and are closely related to ongoing bone destruction and the inflammatory state. These markers are particularly useful for identifying an “inflammatory osteoporosis” subtype [41, 62].

## 6. Therapeutic Opportunities Targeting Neutrophil Activity

As key players in bone‐immune regulation, neutrophils have been gradually regarded as emerging targets for the treatment of osteoporosis in recent years. Their dual contributions, enhancing osteoclast activity and inhibiting osteogenesis, provide multiple avenues for targeted therapy. Several neutrophil‐focused therapeutic strategies are currently being explored.

### 6.1. Inhibiting NET Formation

Given the pivotal role of NETs in osteoporosis pathogenesis, inhibiting NET formation has become an innovative antiresorptive strategy [20]. For example, PAD4 inhibitors that block NETosis, and selective estrogen modulators such as raloxifene, have been shown to reduce NET formation [9, 20].

### 6.2. Targeting Pathogenic Neutrophil Subsets

In the inflammatory and aging bone marrow microenvironment, neutrophils secrete TGF‐β1 and activate the CCR5 signaling axis, which suppresses MSC osteogenesis while promoting osteoclast development, an important mechanism in senile osteoporosis. Therapies targeting the TGF‐β1^+^CCR5^+^ neutrophil subset have been shown to significantly slow bone loss, opening new directions for immune intervention [19].

### 6.3. Neutrophil‐Mediated Drug Delivery

Leveraging the high chemotaxis and bone marrow homing ability of neutrophils, neutrophil‐based drug delivery systems have been developed for bone‐targeted therapy. For instance, “neutrophil‐loaded nanoparticles” can accurately deliver antibone‐resorptive drugs such as teriparatide to the bone marrow, demonstrating excellent targeting and therapeutic effects in animal models [63].

### 6.4. Enhancing Neutrophil Apoptosis

Dysfunctional neutrophils are a driving factor in the chronic inflammatory state of osteoporosis. Restoring neutrophil homeostasis by promoting the clearance of senescent neutrophils is another therapeutic approach. Studies suggest that enhancing the apoptosis of aging neutrophils by modulating the Jak2‐STAT5‐Bax/Mcl‐1 pathway can rebalance the immune environment and alleviate bone destruction [26].

### 6.5. Antioxidant and Anti‐inflammatory Therapies

Because neutrophil‐derived ROS and inflammatory factors significantly contribute to bone resorption, antioxidant therapies have been investigated as adjunct treatments [26]. For example, the natural plant compound andrographolide can reduce neutrophil infiltration and relieve joint inflammation, indirectly reducing the occurrence of secondary osteoporosis in inflammatory conditions [64].

## 7. Discussion and Future Prospects

In recent years, increasing attention has been paid to the role of neutrophils in osteoporosis. Once regarded merely as inflammatory effector cells, neutrophils are now seen as central hubs in the bone immune regulatory network. They are deeply involved in bone metabolism imbalance through multiple mechanisms and play key pathological roles, particularly in PMO and senile osteoporosis.

Neutrophils directly promote osteoclastogenesis by expressing mRANKL or are indirectly activated by various proinflammatory factors (such as IL‐1β, TNF‐α, and CXCL8). Concurrently, releasing ROS and the NETs formed by neutrophils can induce bone marrow macrophages to activate NLRP3 inflammasomes and amplify osteoclast signaling, while also causing osteoblast apoptosis and inhibiting MSC osteogenic differentiation. This “two‐way destructive mechanism” of increasing bone resorption and inhibiting bone formation constitutes the core of neutrophils’ role as an important trigger of osteoporosis [20, 26, 40].

It is also noteworthy that systemic factors such as aging and estrogen deficiency profoundly reshape neutrophil function and phenotypic fate. For example, TCN subsets are enriched in the aged bone marrow; these cells activate osteoclast differentiation while blocking MSC osteogenic differentiation, and they are one of the key pathways driving immunosenescence‐related bone loss [19]. Meanwhile, NETosis activity is significantly enhanced in the PMO mouse model, exacerbating bone resorption through the cGAS‐STING‐AKT2 signaling axis, indicating that NETs can serve as a novel target for immune intervention therapy [20].

At the clinical translation level, neutrophil‐derived blood inflammatory parameters such as NLR and SII, as well as NET components like Cit‐H3, have shown good predictive and diagnostic value as noninvasive biomarkers [41, 48, 50]. Using these markers in combination with traditional bone density tests (e.g., dual‐energy x‐ray absorptiometry) is expected to provide a more effective diagnostic strategy for early screening and accurate assessment of osteoporosis. Moreover, while neutrophil‐derived biomarkers such as NLR, SII, and Cit‐H3 exhibit promising value for risk prediction, stratification, and disease monitoring, their translation into clinical decision‐making is still in an exploratory stage. At present, these indices serve mainly as adjunctive tools to complement imaging‐based assessments rather than as stand‐alone determinants of therapeutic strategies.

In addition, new neutrophil‐targeted therapeutic strategies proposed in recent years, encompassing osteoclast inhibition, osteoblast activation, immune modulation, and novel drug delivery, have emerged as promising avenues for the precision treatment of osteoporosis. For example, the use of PAD4 inhibitors to inhibit NET formation and CCR5 blockers to target aging‐associated neutrophil subsets has shown therapeutic potential in animal models [9, 19]. Likewise, neutrophil‐loaded nanoparticles achieve precise drug delivery through their bone marrow homing characteristics, providing a novel concept for antiresorptive therapy [63]. However, it should be emphasized that neutrophil‐targeted therapies such as PAD4 inhibitors and CCR5 blockers have thus far been evaluated primarily in preclinical and animal models. To date, no clinical trials in humans have been reported, underscoring the urgent need for rigorous translational and clinical studies before these approaches can be integrated into practice.

However, there are still several challenges and limitations in current research. First, the heterogeneity of neutrophils in bone marrow has not been fully elucidated, especially regarding the spatiotemporal evolution of functional subpopulations under different pathological conditions. Second, achieving targeted regulation of neutrophils without impairing their essential immune defense functions requires highly selective intervention strategies. Third, most current evidence is based on animal models and in vitro experiments, and there is a lack of multicenter, large‐sample clinical validation.

Future research should combine cutting‐edge technologies such as single‐cell omics and spatial transcriptomics to analyze the dynamic ecological characteristics of neutrophils in the bone marrow microenvironment and further explore their interaction mechanisms with the bone marrow immune microenvironment, blood vessels, adipocytes, etc., to provide a scientific basis for the formulation of individualized, immune‐targeted osteoporosis intervention strategies.

## 8. Conclusion

Neutrophils, as the most abundant innate immune cells, are being increasingly acknowledged as multifaceted regulators in the pathogenesis of osteoporosis, beyond their traditional role as “inflammatory effector cells.” They not only promote osteoclast differentiation by releasing RANKL and ROS and by forming NETs but also inhibit osteoblast function and the regulation of MSC fate. This leads to a characteristic bone metabolism imbalance with enhanced bone resorption and impaired bone formation.

In PMO and immunosenescence‐related osteoporosis, neutrophils become an important bridge between systemic immune imbalance and local bone destruction due to their high sensitivity to estrogen deficiency, proinflammatory environments, and aging signals. In recent years, the identification of functional neutrophil subsets (such as TGF‐β1^+^CCR5^+^ TCNs) and their activation markers (e.g., CitH3) has provided new perspectives for mechanism research, diagnostic prediction, and precision treatment in osteoporosis.

Clinically, noninvasive biomarkers represented by neutrophil‐related inflammatory indices (such as NLR and SII) and NET components are becoming important tools to complement bone density detection, especially for early screening and disease subtyping. Meanwhile, small‐molecule therapies aimed at inhibiting NETosis, neutrophil‐based drug delivery systems, and immune‐targeted treatments are showing promising therapeutic prospects.

Looking to the future, in‐depth analysis of the dynamic heterogeneity of neutrophils under different pathological conditions, elucidation of their crosstalk with multiple cell types in the bone microenvironment, and the development of highly selective targeted modulation strategies will be key to advancing neutrophil research toward clinical intervention. Neutrophils are expected to become the central hub linking inflammation, immunity, and bone metabolism, providing a breakthrough opportunity for the precise prevention and treatment of osteoporosis.

## Consent

The authors have nothing to report.

## Disclosure

All authors have read and agreed to the published version of the manuscript.

## Conflicts of Interest

The authors declare no conflicts of interest.

## Author Contributions

Conceptualization, methodology, validation, formal analysis, investigation, resources, and data curation: H.C., Y.C., and G.W.; software and writing–original draft preparation: H.C. and Y.C.; writing–review and editing: all authors; visualization: Y.C. and G.W.; supervision and project administration: D.W. and X.L.; H.C. and Y.C. contributed equally to this work.

## Funding

This research received no external funding.

## Data Availability

Data sharing is not applicable. No new data were generated.
